# The Regimental Medical Officer: An Outline of His Duties

**Published:** 1915-10-09

**Authors:** 


### THE REGIMENTAL MEDICAL OFFICER.

### AN OUTLINE OF HIS DUTIES.

A great number of medical men, many of them residents in hospitals and other institutions, are now taking up commissions in the R.A.M.C. who have had no former experience in this branch of their profession, and this number will tend to increase rather than diminish during the next few months, if the medical needs of the Army are to be adequately met. In the usual course of events, newly-commissioned officers of the R.A.M.C. are thoroughly grounded in the routine administration of all classes of work they will be called upon to perform; but in the present emergency there is no time for this instruction of temporarily commissioned medical officers, and they enter on their new duties with only their professional knowledge, which, however complete, cannot guide them in the working of the organisation and administration of the Army Medical Service. This is to some extent overcome in the case of men who are sent to hospitals, or field medical units, as they are there instructed by their own officers in these essentials. Most men will, however, be sent as regimental medical officers to the newly-formed infantry battalions of the New Army, and in this position the freshly-appointed M.O. will have to rely entirely on his own knowledge and initiative in the organisation and carrying out of his work. As we have the authority of the Director-General of the R.A.M.C. himself in stating that the regimental medical officer is the most important unit in preserving the health of the Army, a few words as to the organisation of the medical establishment of a battalion, and the methods by which the work is carried out, ought not to come amiss to those who are likely to be called upon shortly to give their services in such a capacity.

### The Medical and Sanitary Establishments.

The *personnel* a regimental medical officer has at his command consists of (1) the regimental medical establishment, and (2) the regimental sanitary establishment. The regimental medical establishment consists of two orderlies to the M.O. (a lance-corporal and a private), and the regimental stretcher-bearers, usually the regimental band, under command of the band-sergeant. These should number thirty-two—two stretcher parties of four to each company. Four companies usually constitute an infantry battalion. The regimental sanitary establishment consists of about sixteen to twenty men, two or four of whom are told off for "water duties." These are usually also under the command of a sergeant or other non-commissioned officer. The duty of the M.O.'s orderly is to attend the M.O. when seeing the sick, and to assist him generally. The duty of the second orderly is to assist the first and to drive the medical cart and look after the medical equipment when on the march. It is most important that the lance-corporal told off as orderly to the M.O. is smart and intelligent. A tendency will be found to allot to the medical establishment men who have been found to be unfitted to carry out the usual combatant duties of the battalion, and the M.O. will have to ensure that the men told off for his use are of average intelligence, otherwise his work will be rendered extremely difficult, and will not be adequately carried out. This applies most particularly to his own orderlies, and the sergeant in charge of the sanitation squad; it is most important that he should have a reliable man in the latter post. Of course there is no objection to the men of the sanitation squad being told off for this duty on account of some minor defect, such as flat-foot or varicose veins, which prevents their carrying out route marches, etc., but which does not unfit them for sanitation work; but the non-commissioned officer in charge of this squad must be reliable and capable of ensuring that the work ordered by the M.O. is carried out in an efficient manner.

### Orderlies and Stretcher-bearers.

The duty of the stretcher-bearers is, as their name indicates, to carry wounded on stretchers from exposed positions to the regimental aid post, and also to render first aid to the wounded. Stretcher exercises and details of their duties will be found in the official R.A.M.C. training manual, a copy of which should be in the hands of every medical officer.

The duty of the sanitation squad is to attend to the sanitation of the camp generally, and particularly to look after the disposal of excreta and refuse, and to ensure the cleanliness of the kitchens and ablution-places. The water-duty men are told off to look after the water supply. They are kept separate from the rest of the sanitation squad, so that there may be no possible contamination of the water, such as might occur if men connected with the latrines, or who have to deal with an infectious case, also did this water duty. Rules as to camp sanitation, etc., are laid down also in the R.A.M.C. training manual.

Having seen that the correct number of men have been allotted to the medical and sanitary establishments, and that the non-commissioned officers are reliable, the next duty of the M.O. is to ensure that they are properly instructed in their duties and carry them out efficiently.

Perhaps the easiest manner by which it can be explained how this may be done is by sketching an average day's work of a regimental medical officer. It is imagined that the regiment is at a home station, either in camp or billets. The work will, of course, differ abroad, but it is with the duties of the newly appointed medical officer to a battalion training at home that we are now concerned. The first duty of the regimental medical officer is to see the sick. This duty has usually to be performed early in the morning so that the men found fit for duty can go on parade. The men's names, who have to be seen are written on a sick report, and after treating each one of them the M.O. will have to fill up the report as to whether the man is fit for duty, for light duty, etc. No great difficulty will be found in this part of the work, as the M.O. is provided with an outfit of medicines, mostly in tablet form, which will be found to meet the majority of the various minor ailments. Serious cases or those requiring surgical treatment will be sent to hospital, and dental cases to the dentist appointed to carry out such duties. The chief difficulty of the M.O. new to the work will probably be the detection of malingerers and their suitable treatment. Nothing but experience can teach this, however, and he will have to work out his own scheme for their detection and disposal. It is a good plan where there are a great number of sick, and when time is limited, to provide several clinical thermometers, and, the orderlies having been instructed in their use, temperatures can be taken while other cases are being seen and so much time saved. The M.O.'s orderlies will also have to be instructed in first aid, etc., and this can usually be carried out in practice during the morning sick parade. It may be advisable also for the M.O. to instruct his own personal servant in this work, so that he may act as understudy to the orderlies, and as an efficient substitute in their absence. After this parade, returns of inoculations, etc., can be filled up and any necessary correspondence attended to. Then vaccinations and inoculations against typhoid, if any, can be performed.

### The Daily Round.

The M.O. is now free to attend to the general sanitation of the camp or billets. It is as well to have the sanitation squad paraded at regular intervals—first, to ensure that they are all present, and, second, to see that each one is allotted some duty and knows how to carry it out. A few simple lectures on camp sanitation should be given to these men. Information on this and similar points will be found in the R.A.M.C. training manual already mentioned.

After inspecting the sanitation squad the M.O. will dismiss them to their duties, and he will then go round the camp or billets with the non-commissioned officer in charge of the squad and inspect the latrines, kitchens, ablution-places, water supply, and the camp or billets generally as to its, or their, cleanliness and general sanitary condition. He will point out to the non-commissioned officer in charge of the squad any defects which he may observe and order what steps he wishes to be taken for their remedy.

This duty accomplished, the next which devolves on him is the training of the stretcher-bearers. As these men usually constitute the regimental band the afternoon will probably have to be chosen for their medical training, as they will be occupied by their musical duties in the morning. In the training of these men the M.O. will again be guided by the exercises laid down in the R.A.M.C. training manual. They will also have to be given lectures and practical training in first-aid work.

The above are the chief duties to be carried out daily by the regimental medical officer. He will also, of course, have to attend any cases of accident, etc., which occur during the day.

He will, too, be called upon from time to time to give lectures on hygiene, etc., to the officers, non-commissioned officers, and men generally of the battalion.

Some general inspections will also have to be carried out occasionally by him. The chief of these will be an inspection of the non-commissioned officers and men, unclothed, except for shirt and trousers; the shirt open at the neck and the sleeves rolled up, the trousers with the legs rolled up, to ensure that the men maintain their personal cleanliness, that they are not verminous, and that their feet are in a healthy condition. It is as well also to make sure, by an occasional inspection, that each man is in possession of his first field dressing, which should be sewn to one of the bottom corners of his tunic. This is necessary because it will be found that the men have a tendency to use this dressing for any small cut or abrasion which they may sustain. Its proper use is, of course, only as a first dressing to be used when a man is wounded in action.

An occasional inspection of water-bottles is also necessary. The men sometimes leave their bottles half full of water or other liquid and untouched for weeks. They thus get in a filthy condition, and many cases of so-called camp diarrhœa may be traced to this cause.

Of course the M.O. will have to arrange that his work interferes as little as possible with the performance of military duties, and will find that he will have to exercise a considerable amount of tact to have his wishes fulfilled. It will usually be found, however, that when his commanding officer and adjutant realise that he has the best interests of the health of the regiment at heart, he will have their co-operation in any measures which he thinks should be taken to ensure the soundness of the battalion.

The knowledge, too, that he, by carefully carrying out such duties, may be the instrument of saving many lives which would otherwise be wasted and lost to the service of their country ought to recompense him, and be its own reward, for any difficulties or obstacles which he may find in his path.

